# Targeting hematologic malignancies with oncolytic vaccinia virus constructs

**DOI:** 10.1186/2051-1426-1-S1-P226

**Published:** 2013-11-07

**Authors:** Nanhai Chen, Mehmet Kilinc, Qian Zhang, Jason Aguilar, Desislava Tsoneva, Maysam Pessian, Boris Minev, Aladar Szalay

**Affiliations:** 1 Genelux Corporation, San Diego, CA, USA; 2 Radiation Oncology, UCSD, La Jolla, CA, USA; 3 Moores Cancer Center, UCSD, La Jolla, CA, USA; 4 Neurosurgery, UCSD, La Jolla, CA, USA; 5 University of Wurzburg, Wurzburg, Germany

## Background

Oncolytic viruses are a promising approach for cancer therapy. Recent clinical trials proved the safety and anti-tumor activity of several oncolytic viruses in solid tumors. However, very little is known about the potential of the oncolytic viruses to target hematologic malignancies. Careful analysis of the effects of the oncolytic viruses in patients with hematologic malignancies will lead to the development of more specific and effective treatment strategies.

## Methods

We studied the abilities of various vaccinia-based oncolytic virus constructs to target and eliminate leukemic cells derived from patients with several hematologic malignancies. We used vaccinia virus constructs derived from the LIVP strain, LIVP 1.1.1. strain (plaque purified isolate of the nonattenuated LIVP strain), and from the WR strain. The ability of these constructs to infect and amplify in the patients’ leukemic cells and blast cells was studied with fluorescent microscopy, flow cytometry, and plaque assays for viral replication.

## Results

We found that our oncolytic virus constructs infected and killed the patient-derived leukemic cells. The LIVP 1.1.1.-based construct was the most efficient in infecting the leukemic cells, followed by the WR-based construct and the LIVP-based constructs. Flow cytometry data suggested that some of the infected cells have blast-like characteristics. Importantly, virus infection with the oncolytic vaccinia virus constructs correlated with the disease progression in these patients. In all experiments, the viral amplification and cytotoxicity were significantly higher in the leukemic cells than in the control healthy mononuclear cell subsets.

## Conclusions

Our findings suggest the potential of our vaccinia-derived oncolytic virus constructs in targeting different hematologic malignancies. These findings are very relevant to the development of optimized clinical grade oncolytic vaccinia viruses for treatment of hematologic malignancies.

